# p53 activation enhances the sensitivity of non-small cell lung cancer to the combination of SH003 and docetaxel by inhibiting de novo pyrimidine synthesis

**DOI:** 10.1186/s12935-024-03337-x

**Published:** 2024-05-04

**Authors:** Yu-Jeong Choi, Kangwook Lee, Seo Yeon Lee, Youngbin Kwon, Jaehyuk Woo, Chan-Yong Jeon, Seong-Gyu Ko

**Affiliations:** 1 Department of Preventive Medicine, College of Korean Medicine, Kyung Hee University, Seoul, 02447 Korea; 2 Department of Food and Biotechnology, Korea University, Sejong, Korea; 3 Department of Internal Medicine, Yale University, New Haven, CT USA; 4 Department of Korean Medicine, Graduate School, Kyung Hee University, Seoul, Korea; 5 Department of Internal Medicine, College of Korean Medicine, Gachon University, Gyeonggi-Do, Korea

**Keywords:** SH003, Docetaxel, Combination treatment, Non-small cell lung cancer, Pyrimidine biosynthesis, *TP53*, Biomarker

## Abstract

**Background:**

Identifying molecular biomarkers for predicting responses to anti-cancer drugs can enhance treatment precision and minimize side effects. This study investigated the novel cancer-targeting mechanism of combining SH003, an herbal medicine, with docetaxel in non-small cell lung cancer (NSCLC) cells. Also, the present study aimed to identify the genetic characteristics of cancer cells susceptible to this combination.

**Methods:**

Cell viability was analyzed by WST-8 assay. Apoptosis induction, BrdU incorporation, and cell cycle analysis were performed using flow cytometry. Metabolites were measured by LC–MS/MS analysis. Real-time PCR and western blotting evaluated RNA and protein expression. DNA damage was quantified through immunofluorescence. cBioPortal and GEPIA data were utilized to explore the mutual co-occurrence of *TP53* and *UMPS* and UMPS gene expression in NSCLC.

**Results:**

The combination treatment suppressed de novo pyrimidine nucleotide biosynthesis by reducing the expression of related enzymes. This blockade of pyrimidine metabolism led to DNA damage and subsequent apoptosis, revealing a novel mechanism for inducing lung cancer cell death with this combination. However, some lung cancer cells exhibited distinct responses to the combination treatment that inhibited pyrimidine metabolism. The differences in sensitivity in lung cancer cells were determined by the TP53 gene status. *TP53* wild-type lung cancer cells were effectively inhibited by the combination treatment through p53 activation, while *TP53* mutant- or null-type cells exhibited lower sensitivity.

**Conclusions:**

This study, for the first time, established a link between cancer cell genetic features and treatment response to simultaneous SH003 and docetaxel treatment. It highlights the significance of p53 as a predictive factor for susceptibility to this combination treatment. These findings also suggest that p53 status could serve as a crucial criterion in selecting appropriate therapeutic strategies for targeting pyrimidine metabolism in lung cancer.

**Supplementary Information:**

The online version contains supplementary material available at 10.1186/s12935-024-03337-x.

## Background

Various factors such as genetic and metabolic alterations and the tumor microenvironment contribute to tumor heterogeneity, which is the main cause of therapeutic failure and drug resistance. To obtain a detailed rationale for stratifying patients for treatment, it is thus necessary to identify cancer-specific biomarkers that can be used to evaluate and predict the efficacy of cancer treatment. Recently, as a field of cancer treatment, the importance of personalized medicine has been emphasized, providing patients with more accurate and effective therapies based on the genetic and molecular profile of their tumor [1]. This approach helps maximize patient-specific efficacy and reduce side effects. Although many kinds of cancer-targeted drug candidates have been developed to date, research on tumor molecular signatures that can predict drug responses remains highly inadequate.

Tumors reprogram their own metabolism to promote cancer progression. Pyrimidine nucleotide metabolism, which synthesizes building blocks (e.g., nucleic acids) essential for cellular function, is frequently dysfunctional in gastric, breast, and lung cancer, and is closely associated with poor prognosis [2, 3]. Pyrimidine metabolism is classified into two main pathways: de novo and salvage pathways. Most proliferating cells replenish their nucleotide pool by activating the de novo synthetic pathway. Amino acids such as glutamine and aspartic acid as precursors initiate the first synthesis process and each step progresses via the catalytic activity of various enzymes including CAD, DHODH, and UMPS. As therapeutic approaches targeting pyrimidine metabolism, the inhibition of pyrimidine precursors or reduction of enzyme levels has been studied [4]. Although most therapeutic agents have been focused on inhibiting enzymes related to the synthetic pathway, they still show a low response and side effects in clinical trials.

Studies have shown that oncogenes play a pivotal role in regulating metabolic pathways and promoting metabolically targeted drug resistance [5], suggesting that genetic alterations contribute to the metabolic plasticity of cancer. In particular, mutations in TP53, which are present in approximately 30-40% of lung cancer patients, upregulate enzymes involved in pyrimidine biosynthesis, leading to resistance to antimetabolites such as 5-FU [6–8]. Furthermore, Wang et al. demonstrated that mutant TP53 induced the expression of CAD and DHODH in lung adenocarcinoma, which correlated with decreased overall survival of lung cancer patients [9]. Therefore, understanding the aberrant cancer metabolism regulated by P53 mutations may lead to selective drug efficacy based on the cancer metabolic landscape that affects drug responsiveness.

SH003, an herbal mixture of *Astragalus membranaceus* (Am), *Angelica gigas* (Ag), and *Trichosanthes Kirilowii Maximowicz* (Tk), has been reported to have anticancer activity in gastric, breast, prostate, and cervical cancer cells [10–13]. It also showed synergistic anticancer effects when used in combination with chemotherapy drugs such as doxorubicin, paclitaxel, and docetaxel [14–16]. It is also effective in alleviating of docetaxel-induced peripheral neuropathy (DIPN) [17], demonstrating its protective effect against side effects of chemotherapy. In recent research, Jung et al. showed that the combination of SH003 and docetaxel exerts a synergistic effect on lung cancer by inhibiting EGFR signaling [18]. An SH003-docetaxel metabolomic study also revealed that treatment with this combination increases pyrimidine metabolism in exosomes, suggesting that uridine is a putative biomarker in exosomes to evaluate the efficacy of this drug combination [19]. Although research on the mechanism of action of SH003 supports its potential as a new drug for cancer treatment, there is insufficient evidence to predict the selective effect of SH003 according to complex cancer characteristics. In this study, we investigated genetic biomarkers that could predict the efficacy of SH003 and DTX treatment in lung cancer cells. For this purpose, we focused on the cell metabolism altered by the combination treatment and consequently found a synergistic effect occurring through the inhibition of de novo pyrimidine synthesis. Interestingly, the sensitivity to the combination treatment was shown to be determined by the endogenous p53 gene status in lung cancer. These results suggest that p53 is a potent biomarker for evaluating the responsiveness of lung cancer cells to combination therapy of SH003 and DTX.

## Methods

### Cell culture and reagents

Human lung cancer cells (H460, H1703, A549, and H358) were purchased from the Korean Cell Line Bank (Seoul, Korea). H1703 cells were cultured in RPMI-1640 (WelGENE, Gyeongsan, Korea) supplemented with 10% FBS (JR Scientific, CA, USA), 1% penicillin/streptomycin solution (WelGENE, Korea), 4500 mg/L d-glucose, 2 mM l-glutamine, and 10 mM HEPES. The other cancer cells were maintained in RPMI-1640 medium containing 10% Fetal Bovine Serum (FBS), 1% antibiotics, and 2.05 mM l-glutamine (Additional files 1, 2).

Docetaxel and orotate (Sigma-Aldrich Inc., MO, USA) were dissolved in DMSO. UMP (Sigma, USA) and uridine (Thermo Fisher Scientific, USA) were dissolved in D.W. SH003 was provided by Hanpoong Pharm and Foods Company (Jeonju, Republic of Korea). The extraction method for SH003 was as previously described [15].

### Cell viability and apoptosis analysis

Cells were treated with the indicated drugs and then WST-8 solution (Daeillab, Korea) was added to the culture medium at a 1:10 ratio. Cells were incubated for 2 h and the absorbance was measured at 450 nm using an ELISA reader (Molecular Devices, USA) (Additional files 3, 4).

After treatment with SH003 and DTX for 24 h, cells were stained with Annexin V (BD Biosciences, USA) and 7-AAD dye (Sigma, USA) and resuspended in 1 × binding buffer (BD Biosciences, USA) for 15 min. Apoptotic cell death was measured by flow cytometry and analyzed using CellQuest Pro software version 5.2 (Additional file 5).

### BrdU incorporation and cell cycle analysis

Cells were treated with SH003 and DTX and 10 μM BrdU (Sigma, USA) was added to the cell culture medium for 1 h prior to harvest. BrdU-labeled cells were harvested, washed with 1 × HBSS (Thermo Fisher Scientific, MA, USA) to remove unincorporated BrdU, and fixed in 70% EtOH. The cells were denatured in 2 N HCl/0.5% Triton X-100 for 30 min at RT and neutralized by adding to 0.1 M sodium tetraborate (pH 8.5) for 2 min at RT. The cells were then resuspended in PBS containing with 0.5% Tween-20 and 1% BSA, incubated with anti-BrdU (1:50) (Santa Cruz Biotechnology, TX, USA) for 30 min at RT and stained with 10 µg of Goat anti-Mouse IgG-FITC (Sigma, USA) for 30 min. After washing, the cells were stained with 5 µg/mL PI solution containing sodium citrate, RNase A, and NP-40 in PBS for 30 min on ice. Cell cycle distribution and BrdU positive cells were analyzed by flow cytometry (FACSCalibur; BD Biosciences, USA). The results were analyzed using CellQuest Pro software version 5.2 (BD Biosciences, USA). S-phase arrest was represented as the proportion of BrdU-negative cells in S phase.

### Western blotting

Proteins were extracted using 2 × sample buffer (ELPIS Biotech. Inc., Korea), separated on SDS-PAGE, and transferred to a nitrocellulose membrane. They were then incubated with the following primary antibodies overnight at 4 °C: anti-CAD, DHODH, UMPS, UPP1, and UCK2 (Proteintech, USA), and anti-PARP, p53, p-p53, γ-H2AX, H2AX, and p21 (Cell Signaling, USA). The blots were incubated with secondary antibodies, followed by exposure using Pierce ECL Western Blotting Substrate (Thermo Fisher Scientific, USA).

### RNA extraction and quantitative real-time PCR

Total RNA was extracted using R&A-BLUE Total RNA Extraction Kit (Intron Biotechnology, Korea). cDNA was synthesized using the cDNA Synthesis Kit (Takara Biotechnology, China). Quantitative real-time PCR (qRT-PCR) was performed using SensiFAST Probe Hi-ROX Kit (Bioline, USA) on the LightCycler 96 instrument (Roche, Germany). Relative mRNA expression was calculated using the delta-delta Ct method and normalized to GAPDH. The sequences of primers are listed in Table 1.

**Table 1:** PCR primer sequences

Gene	Primer	Sequence (5′–3′)
*DHODH*	Forward	CATAATTGGGGTTGGTGGTG
	Reverse	CTTGGGAAGGTTCCAGATCA
*UMPS*	Forward	GGCTCAGGAGTTGTGAAAGG
	Reverse	CCTGCTTCCAACTGAACTCC
*SLC28A1*	Forward	AGGTCCTGCCCATCATTGTC
	Reverse	CAAGTAGGGCCGGATCAGTA
*SLC28A2*	Forward	AATGGGTGTTTGCAGGAGTC
	Reverse	GAAGACCTAGGCCCGAAAAC
*SLC28A3*	Forward	GACTCACATCCATGGCTCCT
	Reverse	TTCCAGGGAAAGTGGAGTTG
*SLC29A1*	Forward	CTGCTCCCGTGGAATTTTT
	Reverse	GATGCAGGAAGGAGTTGAGG
*SLC29A2*	Forward	CCTCCTTCCCTGGAACTTCT
	Reverse	GTTGAGGAGGGTGAAGAGCA
*SLC29A3*	Forward	GCCAACTTCCTGCTTGTCA
	Reverse	GTGCCTGGGAGTTCCTCATA

### Transfection

Cells were transfected using Lipofectamine RNAiMAX reagent or Lipofectamine 3000 (Thermo Scientific, USA). p53 siRNA was obtained from Santa Cruz (sc-29435, USA) and p53 plasmid was purchased from Addgene (#69003). The UMPS siRNA (5′-GAGAACCACTTCACTGGTT-3′) was synthesized by Bioneer (Daejeon, Korea). Further experiments were performed at 72 h post-transfection.

### Immunofluorescence

Cells were fixed with 4% paraformaldehyde (PFA) for 10 min and permeabilized with 0.5% Triton X-100 for 7 min. Cells were washed in PBS and blocked with blocking buffer (PBS with 10% FBS, 1% BSA, and 0.1% Tween-20) for 1 h at RT. Primary antibody γ-H2AX (1:300) was applied overnight at 4℃. Fixed cells were incubated with Alexa Fluor 488-conjugated anti-goat IgG (1:300) for 1 h at RT, and counterstained with 1 µg/mL DAPI (Sigma, USA). Representative images were obtained using a confocal microscope (Zeiss LSM 900; Carl Zeiss, Germany).

### Quantification of metabolites

For targeted metabolomic studies, cell lysates were mixed at a 1:2 ratio with 100% methanol, vortexed, and centrifuged at 15,000 rpm at 4 ℃ for 10 min. The supernatant was obtained and analyzed by LC–MS. Samples were injected on a Poroshell 120 EC-C18, 2.1 × 100 mm, 2.7 µm column (Agilent Technologies, USA). The samples were separated by gradient elution using solvent A (0.1% formic acid + 5 mM ammonium formate in water) and solvent B (0.1% formic acid in methanol). The gradient was as follows: 0–0.1 min with 1% solvent B, 0.1–7 min with 1% solvent B, 7–7.1 min with 70% solvent B, and 7.1–10 min with 1% solvent B. Mass spectrometry (MS) was performed in positive ion mode (Agilent Jet Stream, Agilent Technologies, USA) with the following conditions: gas temperature 290 ℃, gas flow 11 L/min, nebulizer pressure 30 psi, sheath gas temperature 350 ℃, and sheath gas flow 11 L/min. The samples were scanned based on the mass-to-charge ratio and the concentration of the orotate and uridine metabolites was calculated using a standard curve.

### Genomic data

To identify the tendency for the TP53 and UMPS genes to be co-mutated in NSCLC, we employed cancer genomic data using cBioPortal (http://cbioportal.org) [20]. A total of 2878 samples from the following seven studies on NSCLC were analyzed: Non-small cell lung cancer (MSK (Science 2015), University of Turin (Lung Cancer 2017), TRACERx (NEJM & Nature 2017), MSKCC (J Clin Oncol 2018), MSK (Cancer Cell 2018), MSKCC (Cancer Discov 2017), and Pan-Lung Cancer (TCGA, Nat Genet 2016).

Comparative data on gene expression in lung cancer and in normal tissue were obtained from Gene Expression Profiling Interactive Analysis (Gene Expression Profiling Interactive Analysis). Boxplots of *UMPS* gene expression present such expression as the log_2_ (TPM + 1), and compared with TCGA and GTEx normal database.

### Statistical analysis

Data are presented as mean ± standard deviation (SD). Statistical analysis was performed with GraphPad Prism software 8.0.2, using one-way ANOVA with Tukey’s test for group comparisons and Pearson’s correlation coefficient (r) for correlation analyses.

## Results

### The combination of SH003 and DTX induces apoptosis by inhibiting the de novo pyrimidine synthesis pathway

**Fig. 1:**
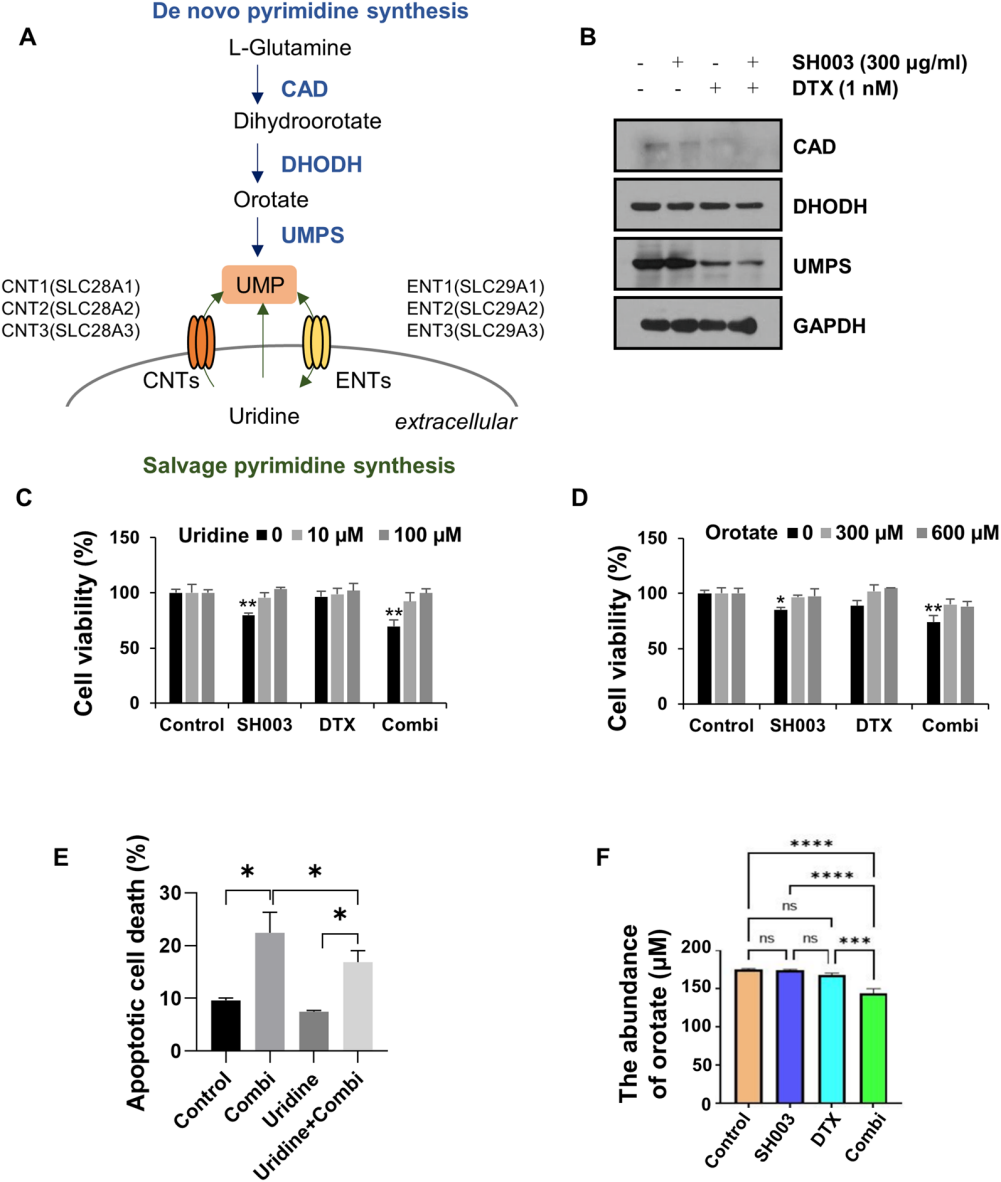
The combination of SH003 and DTX induces apoptosis by blocking de novo pyrimidine synthesis in H460 cells. **A** Schematic of pyrimidine metabolism. **B** H460 cells were treated for 24 h with SH003 and/or DTX. The expression of enzymes involved in de novo pyrimidine synthesis was measured by western blotting. **C**, **D** Cells were treated with the combination of SH003 and DTX for 1 h and then incubated with uridine or orotate at the indicated doses for 24 h. Cell viability was analyzed using WST-8 assay. **E** After treatment with the combination for 1 h, cells were treated with 100 μM uridine for 24 h and the increase in apoptosis was determined by flow cytometry. **F** The concentration of orotate was measured by LC–MS in H460 cells treated with the combination for 24 h. *p* value was calculated by one-way ANOVA with Tukey’s test. *p < 0.05; **p < 0.01; ***p < 0.001

**Fig. 2:**
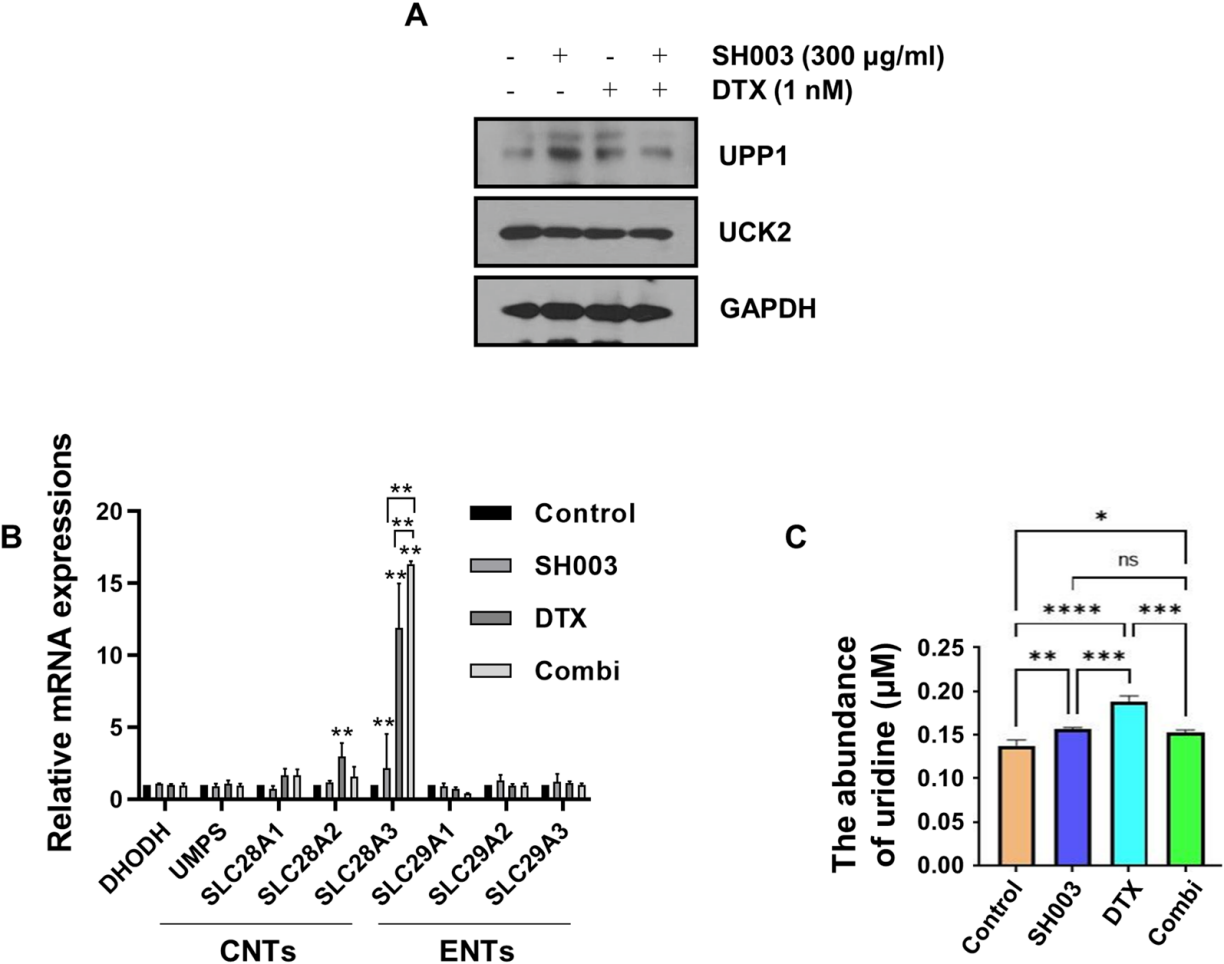
The influx of uridine is activated to replenish pyrimidine pools blocked by the combination in H460 cells. **A** The expression of enzymes involved in the salvage pathway. **B** qRT-PCR for DHODH, UMPS, and human nucleotide transporters in H460 cells after combination treatment. *p* value was calculated by one-way ANOVA with Tukey’s test. **C** The concentration of uridine was analyzed by LC–MS. *p < 0.05; **p < 0.01; ***p < 0.001. *CNT* concentrative nucleoside transporter, *ENT* equilibrative nucleoside transporter

Cancer cells rely on the de novo synthesis of pyrimidine nucleotides for uncontrolled proliferation or an abundant energy supply [7]. Our previous study involving metabolic profiling analysis in lung cancer cell-derived exosomes showed that combination treatment of SH003 and DTX regulates pyrimidine metabolism [19]. Therefore, to determine whether the combination of SH003 and DTX regulates pyrimidine metabolism in lung cancer cells, we tested the expression of enzymes involved in the production of UMP, a nucleotide generated for in the process of pyrimidine biosynthesis, in combination-treated H460 lung cancer cells. Our previous study identified concentrations of SH003 and DTX (300 µg/mL SH003 and 1 nM DTX) that exert synergistic effects in H460 and A549 lung cancer cells [18], so further experiments were performed with these concentrations. Figure 1A briefly depicts the procedure for UMP synthesis. The combination treatment inhibited the expression of CAD, DHODH, and UMPS enzymes that catalyze de novo UMP production (Fig. 1B). However, the levels of mRNAs that encode these proteins did not differ (Fig. 2B), indicating the post-translational modification of enzymes by the combination treatment. Next, to investigate the role of the pyrimidine biosynthesis pathway in the cell death induced by the combination treatment tested in this study, cell viability was measured in the presence of uridine and orotate, which are intermediate metabolites of UMP synthesis. The effect of the combination treatment on cell viability was fully restored by the addition of both uridine and orotate (Fig. 1C, D). The pro-apoptotic effect of SH003 and DTX was also inhibited by uridine (Fig. 1E), suggesting that pyrimidine metabolism is related to the mechanism by which SH003 and DTX induce the death of lung cancer cells. Consistent with this, the combination treatment inhibited the production of orotate in H460 cells (Fig. 1F), confirming that SH003 and DTX impaired de novo pyrimidine synthesis.

### Inhibition of de novo pyrimidine synthesis by combined treatment accompanies intracellular uridine accumulation

Another pyrimidine synthesis pathway, the salvage pathway, introduces nucleosides such as cytidine and uridine from the extracellular environment into the cell, helping to maintain the UMP pool at a constant level [21]. To test whether the combination of SH003 and DTX inhibits not only the pyrimidine biosynthesis but also the pyrimidine salvage pathway, we determined the levels of UCK2 and UPP1 enzymes, which phosphorylates uridine or uracil nucleosides to form UMP as salvage pathways [22]. Neither enzyme showed much change in cells treated with the combination (Fig. 2A), showing that this combination does not participate in the enzymatic reaction of the salvage pathway. Nucleoside transport pumps are also responsible for replenishment of the UMP pool through the recycling of extracellular nucleosides. Among members of the nucleoside transporter family, the mRNA expression of the concentrative nucleoside transporter, CNT3 (SLC28A3), was increased by the combined treatment (Fig. 2B). We also found that the intracellular level of uridine was enhanced upon SH003 and/or DTX treatment (Fig. 2C). Although SH003 and DTX induced apoptotic cell death by inhibiting the de novo pyrimidine synthesis pathway, they simultaneously activated the nucleotide salvage pathway. These results suggest that uridine accumulation can be considered as a cellular homeostatic mechanism to restore the imbalance of pyrimidine synthesis caused by SH003 and DTX treatment.

### NSCLC is selectively sensitive to UMPS enzyme inhibition by combination treatment

**Fig. 3:**
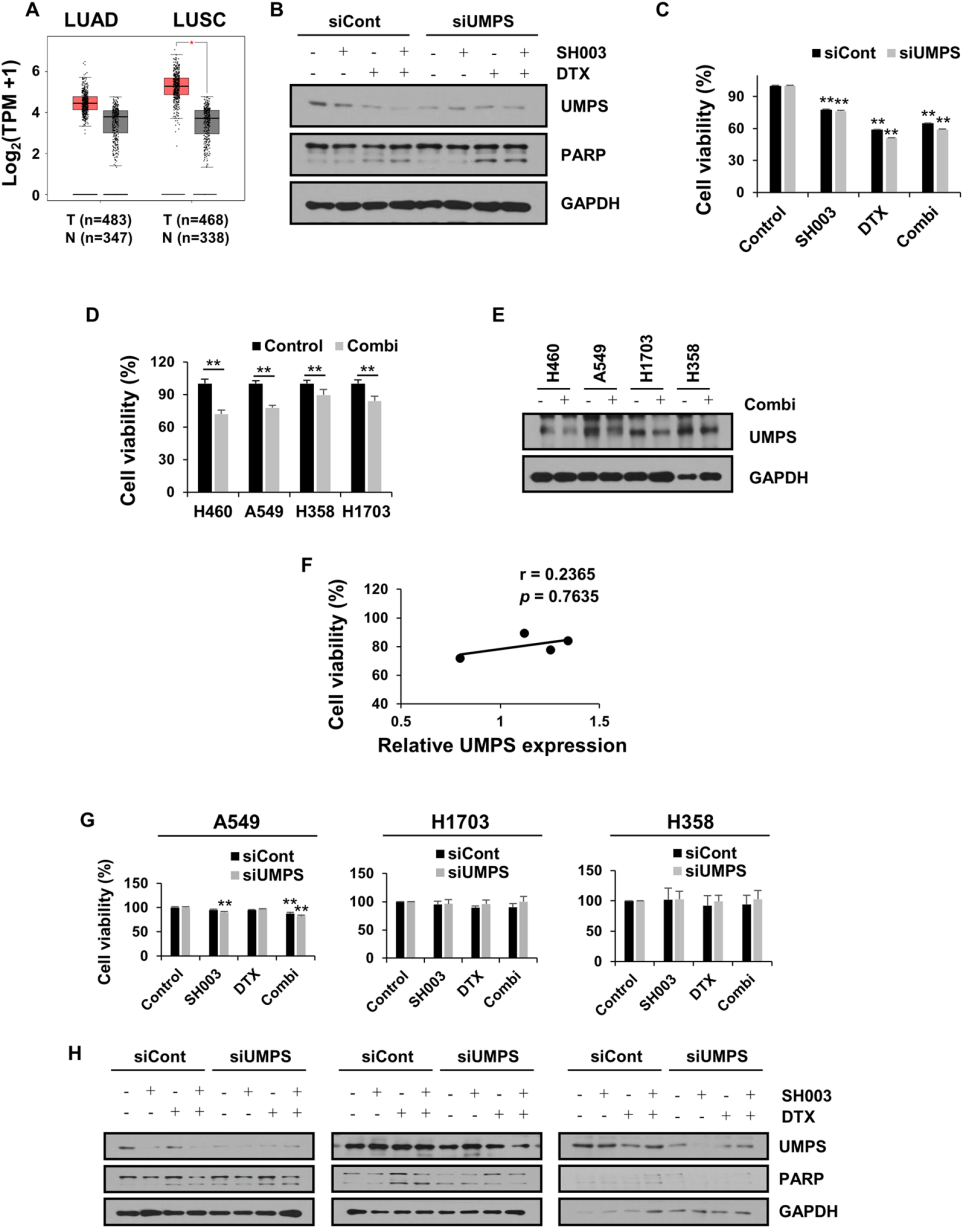
Targeting of the UMPS enzyme by the combination treatment has a selective inhibitory effect on NSCLC cells. **A** Boxplot indicates the UMPS expression in LUAD, LUSC and normal tissue. The different gene expression data were obtained from the GEPIA tool and analyzed using matched data in TCGA normal and GTEx. Red boxplot: Tumor group (T), grey boxplot: Normal group (N). *p < 0.01. **B**, **C** Upon UMPS knockdown in H460 cells, the expressions of UMPS and the apoptosis marker; PARP and cell viability were determined following combination treatment for 24 h. **D** Cell viability was measured after treatment with 100 μg/mL SH003 and 1 nM DTX for 24 h in H460, A549, H358, and H1703 NSCLC cell lines. **E** Western blotting of UMPS levels in combination treated-lung cancer cells at 24 h. **F** The relationship between cell viability (**D**) and UMPS levels (**E**) shown in NSCLC cells was evaluated by Pearson’s r using GraphPad Prism software. **G**, **H** Cell viability and protein expression upon SH003 and/or DTX treatment in UMPS deleted cells. Statistical significance was determined by Student’s t test or one-way ANOVA with Tukey’s test. *p < 0.05; **p < 0.01

UMPS is the final enzyme for UMP biosynthesis and also functions as an oncometabolite [23, 24]. In gene expression analysis by GEPIA, UMPS expression in NSCLC patient tissues was higher than in normal tissues, which exhibited a particularly significant difference in lung squamous cell carcinoma (LUSC) (Fig. 3A). We therefore hypothesized that the inhibition of UMPS is essential for the action of the pyrimidine-targeting combination on NSCLC. We first evaluated the inhibitory effect of UMPS on combination-induced cell death in UMPS-silenced H460 cells. UMPS knockdown enhanced the apoptotic and cytotoxic effects of SH003 and DTX (Fig. 3B, C), suggesting that the induction of apoptosis by this combination is mediated by UMPS inhibition. To investigate whether other NSCLC cells have the same efficacy as shown by these results, we performed further tests on A549, H358, and H1703 lung cancer cells. The combination treatment inhibited all tested lung cancer cells, but reduced cell viability more effectively in H460 and A549 cells than in H358 and H1703 cells (Fig. 3D). In addition, upon co-treatment with SH003 and DTX, UMPS levels were decreased in all NSCLC cells (Fig. 3E), but there was no significant correlation between UMPS expression and the sensitivity of NSCLC cells to the combination (Fig. 3F). These results indicated that the inhibition of UMPS does not directly affect the response to the combination treatment. Interestingly, upon the inhibition of UMPS, the apoptotic effect of the combination was specifically enhanced in lung cancer cells (H460 and A549 cells) that were more sensitive to the combination, while the apoptotic cell death of H1703 and H358 cells remained unaltered (Fig. 3G, H). Collectively, these results suggest that the inhibition of UMPS by SH003 and DTX treatment is not a key factor in targeting all lung cancer types, and that a clearer molecular target needs to be identified to predict the effect of the combination.

### Susceptibility of p53 wild type lung cancer cells to the combination involves UMPS inhibition-mediated apoptosis

**Fig. 4:**
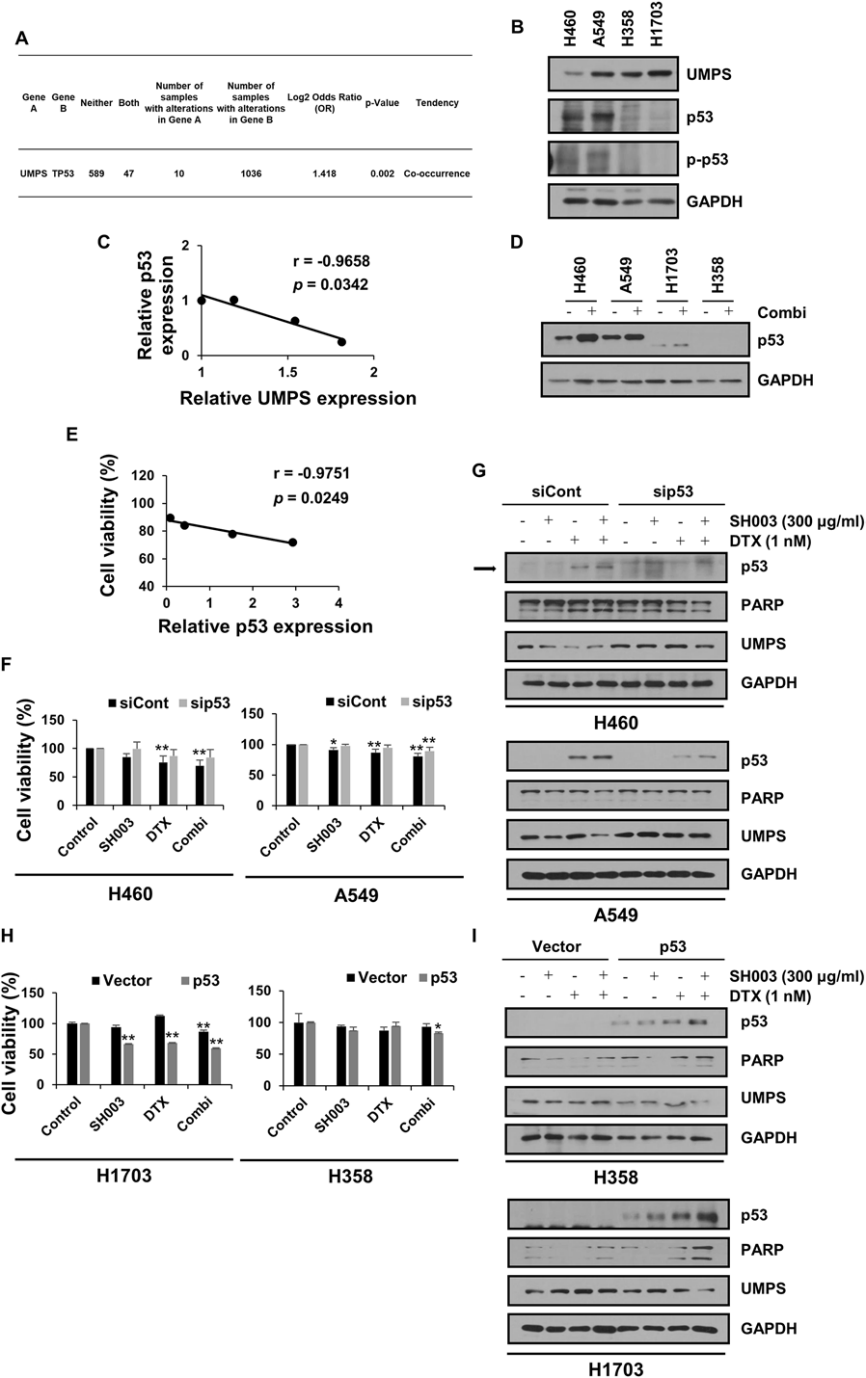
p53 upregulation determines the action of the combination through UMPS inhibition-mediated apoptosis in only NSCLC with wild type p53. **A** The tendency for co-occurrence of mutant *TP53* and UMPS tested in NSCLC patients in seven datasets. **B** The basal expression levels of UMPS and p53 in NSCLC. **C** The correlation between p53 and UMPS expression in NSCLC. **D** NSCLC was treated with the combination of SH003 and DTX for 24 h and the expression of p53 was determined by western blotting. **E** The correlation of p53 protein levels and cell viability was changed by the combination in NSCLC. **F**, **G** H460 and A549 cells were treated with SH003 and/or DTX for 24 h following p53 knockdown. **H**, **I** H1703 and H358 cells were treated with SH003 and/or DTX for 24 h following p53 overexpression. The cell viability and protein expression levels were measured. Statistical significance was determined by one-way ANOVA with Tukey’s test. *p < 0.05; **p < 0.01

To identify a biomarker that determines the sensitivity to the combination tested in this study, the genetic variations in lung cancer cell lines were considered and differences in *TP53* gene features were identified between the cells as follows: H460 and A549 (p53 wild type), H1703 (p53 mutant), and H358 (p53 null) cell lines. Moreover, genomic profiling of NSCLC patients from the cBioPortal database revealed the co-occurrence of gene mutations in TP53 and UMPS (Fig. 4A), suggesting that p53 mutation may affect UMPS function. Therefore, we investigated whether p53 expression would affect the apoptotic mechanism of the combination targeting UMPS. Interestingly, endogenous p53 and UMPS expression levels in the four NSCLC cell lines showed a negative correlation (Fig. 4B, C). In addition, the level of p53 protein was increased more in H460 and A549 cells than in H1703 cells after combination treatment, while its expression was not detected in H358 cells (Fig. 4D). These changes in p53 levels were significantly associated with decreased viability of NSCLC cells (Fig. 4E), indicating that the upregulation of p53 in cells harboring wild type p53 is closely related to the sensitivity to combination treatment. Next, we evaluated whether p53 is a key mediator of the anti-cancer effect of the combination through the suppression of UMPS. Knockdown of p53 in p53 wild type lung cancer cells (H460 and A549 cells) prevented the combination’s effect on cell growth (Fig. 4F). In addition, the combination-induced cleavage of PARP was blocked by p53 inhibition, and UMPS expression was also restored (Fig. 4G). Meanwhile, when normal p53 was transfected in both p53-mutant H1703 and -deficient H358 cells, sensitivity to the combination treatment was improved, especially in H1703 cells (Fig. 4H), accompanied by UMPS inhibition and induction of PARP cleavage (Fig. 4I). These findings suggest that p53 initiates combination-induced apoptosis via the inhibition of pyrimidine metabolism, and thus drug sensitivity depends on the genetic status of *TP53* in NSCLC.

### Inhibition of pyrimidine nucleotide biosynthesis by combination treatment leads to DNA damage by activating p53

**Fig. 5:**
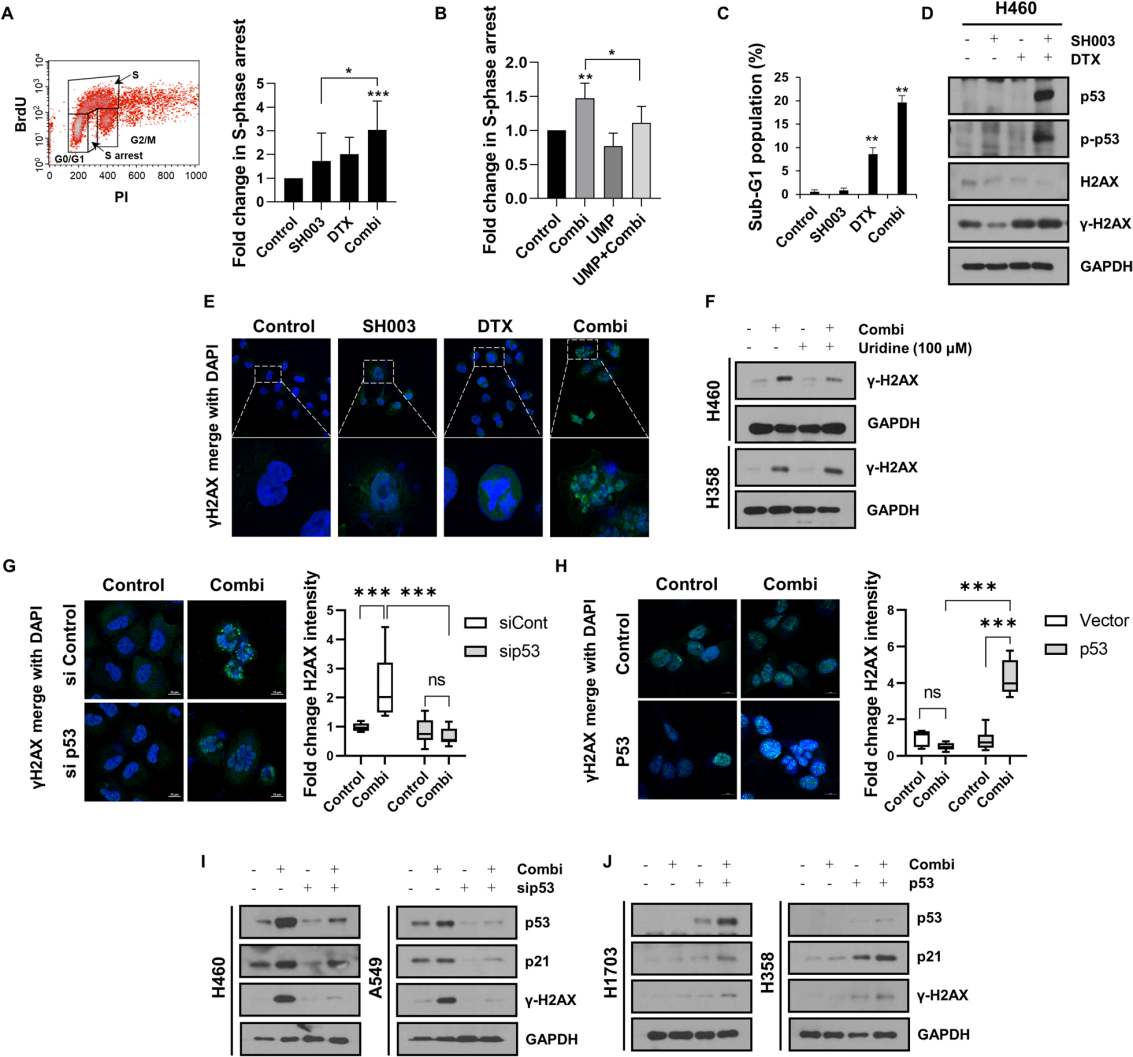
Combination treatment-mediated p53 activation induces replication stress and DNA damage through reduction of the UMP pool in only H460 cells. **A** H460 cells were treated with the combination for 6 h and stained with anti-BrdU. BrdU-negative cells in S phase were analyzed by flow cytometry. The flow cytometry image shows one representative result. **B** Bar graph indicates the proportion of cells in S-phase arrest. **C** H460 cells were treated with SH003 and/or DTX for 24 h, and the cell cycle was analyzed by staining with PI. Histogram represents sub-G1-phase cells. **D** Western blot analysis of a DNA damage marker, γ-H2AX, and p-p53. **E** Immunofluorescence analysis of γ-H2AX in the cell nuclei after exposure to SH003 and/or DTX for 24 h in H460 cells. **F** Cells were treated with the combination followed by uridine for 24 h. The expression of γ-H2AX protein was analyzed by western blotting. **G** H460 cells were treated with SH003 and DTX for 24 h following p53 knockdown, fixed, and incubated with anti-γ-H2AX. The intensity of γ-H2AX foci was calculated using Image J. **H** Confocal image indicated the formation of γ-H2AX foci in p53-overexpressing H358 cells. Bar graph showed the relative intensity of γ-H2AX foci. **I** Upon the transfection of H460 and A549 cells with sip53, the protein levels of p21 and γ-H2AX were detected by western blotting. **J** Upon the overexpression of p53 vector in H358 and H1703 cells, the protein levels of p21 and γ-H2AX were determined by western blotting. Statistical significance was determined by one-way ANOVA with Tukey’s test. *p < 0.05; **p < 0.01

**Fig. 6:**
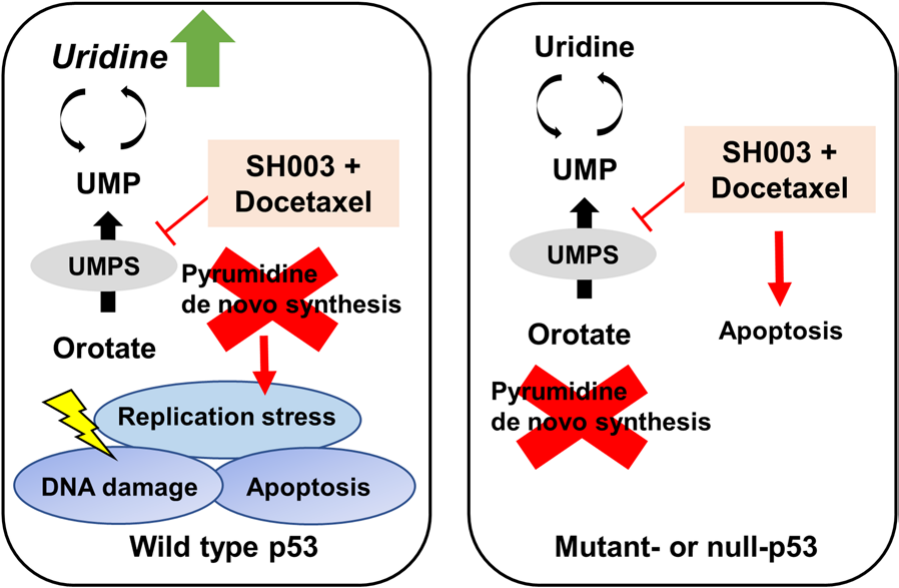
A schematic of the mechanism by which the combination of SH003 and DTX induces NSCLC cell death. Treatment with SH003 and DTX inhibited the de novo pyrimidine synthesis pathway by activating p53 in p53 wild type NSCLC cells. The reduction of pyrimidine synthesis-related UMPS enzyme levels in turn caused replication stress, DNA damage, and apoptosis. In NSCLC cells harboring p53 mutation or null type, combination treatment induced apoptosis independent of p53-mediated metabolic perturbations

The blockade of intracellular nucleotide synthesis induces replication stress, which is a cause of DNA damage [25]. p53 is activated in response to excessive DNA damage and then initiates apoptosis signal transduction by inducing transcription of apoptosis related genes [26]. To investigate how the increase in p53 due to the combination regulates apoptosis by reducing pyrimidine synthesis in NSCLC cells, we first examined the impact of combination treatment on replication stress and DNA damage response. We found that BrdU^low^/S-phase cells, undergoing S-phase arrest referred to as replication stress, were increased in combination-treated H460 cells (Fig. 5A). In addition, the replication stress in combination-treated cells was restored in the presence of UMP (Fig. 5B), indicating that co-treatment disrupts DNA replication by inhibiting pyrimidine biosynthesis. Next, we analyzed the distribution of sub-G1 cells to identify the apoptotic cells with fragmented DNA. Combination treatment induced the accumulation of sub-G1 populations in H460 cells (Fig. 5C). In addition, the expression of γ-H2AX, a DNA damage marker, was increased upon combination treatment, along with p53 phosphorylation in H460 cells (Fig. 5D). Moreover, the formation of γ-H2AX foci in the nucleus was also observed in combination-treated cells (Fig. 5E), suggesting the DNA damage-inducing effect of the combination. Notably, uridine supplementation reduced γ-H2AX expression induced by the combination only in p53 wild type H460 cells, but it was unchanged in p53 null-type H358 cells (Fig. 5F), indicating that the inhibition of pyrimidine synthesis after co-treatment causes p53-mediated DNA damage response. We confirmed the ability of p53 to induce DNA damage in p53-knockdown or -overexpressing cells. In p53-knockdown H460 cells, the formation of γ-H2AX foci caused by combination treatment was reduced (Fig. 5G). Meanwhile, in p53-overexpressing H358 cells, γ-H2AX foci were significantly increased by combination treatment (Fig. 5H). In line with this, we also found that the increases in expression of γ-H2AX and p21, p53 target genes dependent on p53 activity, by the combination were blocked by p53 knockdown in both p53 wild type cells (Fig. 5I). In contrast, when wild type p53 is expressed in p53 mutant or null cells, combination treatment resulted in the upregulation of γ-H2AX and p21 (Fig. 5J). These results suggest that the mechanism behind the combination’s effect on DNA damage-induced apoptosis in NSCLC is determined by the normal transcriptional activity of p53. Taking these findings together, the activation of p53 by the combination treatment specifically in p53 wild type NSCLC cells resulted in the disruption of pyrimidine nucleotide synthesis, which induced replication stress and DNA damage as stimulators of apoptosis. Therefore, the mechanisms by which the combination inhibits NSCLC can be classified into two signaling pathways depending on the genetic status of p53: DNA replication stress-dependent or independent apoptotic cell death pathways (Fig. 6).

## Discussion

Previous research has shown that SH003 not only exerts anti-cancer effects, but also acts synergistically with DTX in NSCLC [18]. However, the detailed mechanism of cancer treatment of SH003 and DTX, which could predict the efficacy of combination treatment and help therapeutic decision-making for patients, has not yet been elucidated. Therefore, there is a need to identify molecular markers that can be targeted by this combination treatment in lung cancer. Here, we demonstrate, for the first time, that the SH003/DTX combination treatment induces apoptosis by inhibiting the UMP biosynthetic pathway involved in pyrimidine metabolism. However, this effect was not reproduced in all lung cancer cells tested. We assumed that this difference in reactivity to the combination targeting pyrimidine metabolism would be determined by the genetic background of lung cancer cells. Indeed, we demonstrated that lung cancer cells with wild type p53 are more susceptible to the metabolic stress induced by the combination compared to those with mutant p53.

Dysfunction in the pyrimidine synthesis process has been identified as a contributor to abnormal cancer progression, making pyrimidine metabolism an attractive target for cancer therapy. Nucleotide analogs like gemcitabine and fluorouracil are commonly used in lung cancer treatment. However, mutations in genes associated with the transport or targets of these drugs within tumors can lead to drug resistance [27]. As part of another treatment strategy for cancer metabolism, most research have been concentrated inhibiting enzymes related to UMP synthesis, with a particular focus on drugs targeting DHODH (e.g., leflunomide) and CAD enzymes [28, 29]. However, clinical trials with these enzyme inhibitors have revealed limitations, including drug tolerance and high toxicity in normal cells [30, 31]. Therefore, future research could explore novel combination of inhibitors to address these challenges. In addition, there has been little study regarding UMPS inhibitor in lung cancer treatment. Our results demonstrated that combined treatment suppresses the UMP synthesis pathway by reducing the protein expression of CAD, DHODH, and UMPS enzymes, along with the accumulation of orotate, an intermediate UMP metabolite. These results suggest the potential of combination treatment in targeting multiple metabolic pathways, thereby augmenting its anti-cancer effects. Notably, the inhibiting UMPS expression in p53 wild-type lung cancer cells enhanced their responsiveness to the combination treatment, supporting the significance of UMPS as an effective target for suppressing lung cancer metabolism.

Uridine, found in the blood, has been regarded as a metabolite with a multifaceted role in cancer therapy. Firstly, uridine serves to counteract the effects of pyrimidine biosynthesis inhibitors by replenishing UMP through the salvage pathway [22]. Consequently, clinical trials have explored combination strategies that simultaneously inhibit nucleoside transporters in the salvage pathway alongside the use of pyrimidine biosynthesis inhibitors. Secondly, uridine has the potential to mitigate side effects induced by pyrimidine metabolism-targeting drugs, such as neurological deficits and myelotoxicity [32, 33]. Our study revealed that the combination treatment, while inhibiting pyrimidine biosynthesis and inducing apoptosis, also led to an increase in intracellular uridine levels. Intriguingly, this rise in uridine did not appear to affect the apoptotic effects of the combination treatment. We additionally observed that the combination treatment stimulated the expression of the SLC28A3 pump, which is associated with increased intracellular uridine levels. Consequently, we inferred that uridine levels increased as a result of activation of the pyrimidine salvage pathway, aligning with its first aforementioned function. Furthermore, we proposed that the regulation of the nucleoside transporters, including SLC28A3, may serves as one of the compensatory mechanisms in response to pyrimidine inhibition. This hypothesis is supported by our prior findings, which indicated that uridine could serve as an endpoint biomarker independently of the synergistic action of the combination treatment [19]. This suggests that an increase in extracellular uridine levels could potentially predict the synergistic effect of the SH003 and DTX combination. However, our study does not comprehensively explore the functional significance of uridine accumulation. Nonetheless, it is crucial to acknowledge that our study did not comprehensively explore the functional significance of uridine accumulation. Therefore, further research is warranted to elucidate the precise mechanisms governing uridine restoration by nucleoside transporters and to establish appropriate thresholds for uridine levels, aiding in the assessment of the response to SH003/DTX combination treatment. It's also important to investigate if the increased uridine levels induced by the combination treatment function as an intracellular countermeasure, mitigating cellular damage resulting from reduced pyrimidine metabolism, as the latter function of uridine.

We also demonstrated that the inhibition of UMPS-mediated cell death induced by the combination treatment is determined by the genetic status of p53 in lung cancer cells, indicating the importance of p53 in UMPS-dependent metabolism. Indeed, oncogene-driven metabolic reprogramming has been shown to significantly influence the response to antimetabolite drugs in cancer therapy [5]. For instance, oncogenes like MYC, KRAS, and mTOR can upregulate the expression of proteins involved in de novo pyrimidine synthesis [9, 34]. Moreover, Iorio et al. have reported that *TP53* mutations can serve as predictive markers for evaluating sensitivity to metabolism-antagonist chemotherapies [35]. It is worth noting that TP53, a well-known tumor suppressor gene, can act as an oncogene when mutated and contributes to de novo pyrimidine synthesis. Consequently, this upregulation of pyrimidine de novo synthesis in cancer cells leads to resistance against antimetabolite drugs. These findings highlight the interplay between genetic features and metabolism of cancer cells. While previous gene expression analyses have indicated that UMPS is a prognostic marker in NSCLC [36, 37], studies understanding the signaling pathway between UMPS-p53 are still lacking. Our analysis of TCGA data revealed a significant co-occurrence of UMPS and p53 mutations in lung cancer (Fig. 3E). Furthermore, this study identified a correlation between the suppression of UMPS expression and increased p53 expression in lung cancer cells. However, the inhibitory effect of the combination treatment on pyrimidine metabolism was insufficient to induce apoptosis in both p53-mutant and p53-deficient lung cancer cells. This suggests that p53 activation is crucial for the sensitivity of lung cancer cells to the combination treatment targeting pyrimidine metabolic pathways. In p53 wild-type lung cancer cells, the combined treatment effectively blocked the pyrimidine pool through the transcriptional activation of p53, subsequently inducing apoptosis mediated by replication stress and DNA damage [38]. Therefore, this study establishes that the combination treatment acts specifically on p53 wild-type lung cancer cells through the inhibition of the pyrimidine de novo mechanism by p53 activation. These findings emphasize the importance of understanding the mutational characteristics of tumors for predicting the response to cancer metabolism-disrupting drugs in a clinical context.

p53-targeted therapeutic strategies are typically categorized into two approaches: those aimed at restoring the function of mutant p53 to that of normal p53 and those focused on enhancing the stability of wild-type p53 function. These strategies activate the normal p53-dependent cell death pathway, such as apoptosis, by stimulating interactions with p53 target genes [39, 40]. However, because these drugs need to address a wide range of p53 gene mutations, achieving selective effects in patients can be challenging [41]. In addition, despite the discovery of numerous p53-based drugs, p53 genetic mutations have been considered as genetic biomarkers for predicting treatment response, as outlined in the National Comprehensive Cancer Network (NCCN) clinical practice guidelines for the treatment of patients with p53 mutant cancers, including lung cancer, breast cancer, and acute myeloid leukemia [42]. Similarly, our results confirmed that the efficacy of combination is determined by the presence or absence of p53 mutations. Hence, it is possible to identify a subgroup of lung cancer patients for whom the combination treatment of SH003 and DTX will be effective by analyzing the p53 expression pattern.

## Conclusion

This study demonstrated that the inhibition of pyrimidine biosynthesis pathways represents a novel mechanism through which the combination treatment of SH003 and DTX exerts its effects on lung cancer cells. In particular, the inhibition of UMPS enzymes by this combination treatment was identified as one of the most effective strategies for inducing the apoptosis in lung cancer cells. Notably, the impact of this combination on pyrimidine metabolism was found to be contingent on the genetic status of *TP53* in non-small cell lung cancer. This strongly suggests that tailoring the combination treatment to match the specific genetic characteristics of tumors holds the potential for achieving more precise and effective anticancer outcomes. In conclusion, the analysis of p53 mutations in lung cancer can aid in the selection of more suitable candidates for the combined treatment of SH003 and DTX against lung cancer.

### Supplementary Information

**Additional file 1.** Uncropped western blot band in Figure 1

**Additional file 2.** Uncropped western blot bands in Figure 2

**Additional file 3.** Uncropped western blot bands in Figure 3

**Additional file 4.** Uncropped western blot bands in Figure 4

**Additional file 5.** Uncropped western blot bands in Figure 5

## Data Availability

The data presented in this study are available in article.

## Abbreviations

DTX: Docetaxel
Am: *Astragalus membranaceus*
Ag: *Angelica gigas*
Tk: *Trichosanthes Kirilowii Maximowicz*
DIPN: Docetaxel-induced peripheral neuropathy
qRT-PCR: Quantitative real-time PCR
PFA: Paraformaldehyde
GEPIA: Gene Expression Profiling Interactive Analysis
SD: Standard deviation
CNT: Concentrative nucleoside transporter
NCCN: National Comprehensive Cancer Network
