# Supplementary figures and images for: p53 activation enhances the sensitivity of non-small cell lung cancer to the combination of SH003 and docetaxel by inhibiting de novo pyrimidine synthesis
Source: Cancer Cell Int. 2024 May 4;24:156. doi: 10.1186/s12935-024-03337-x (PMC11069295; doi:10.1186/s12935-024-03337-x)

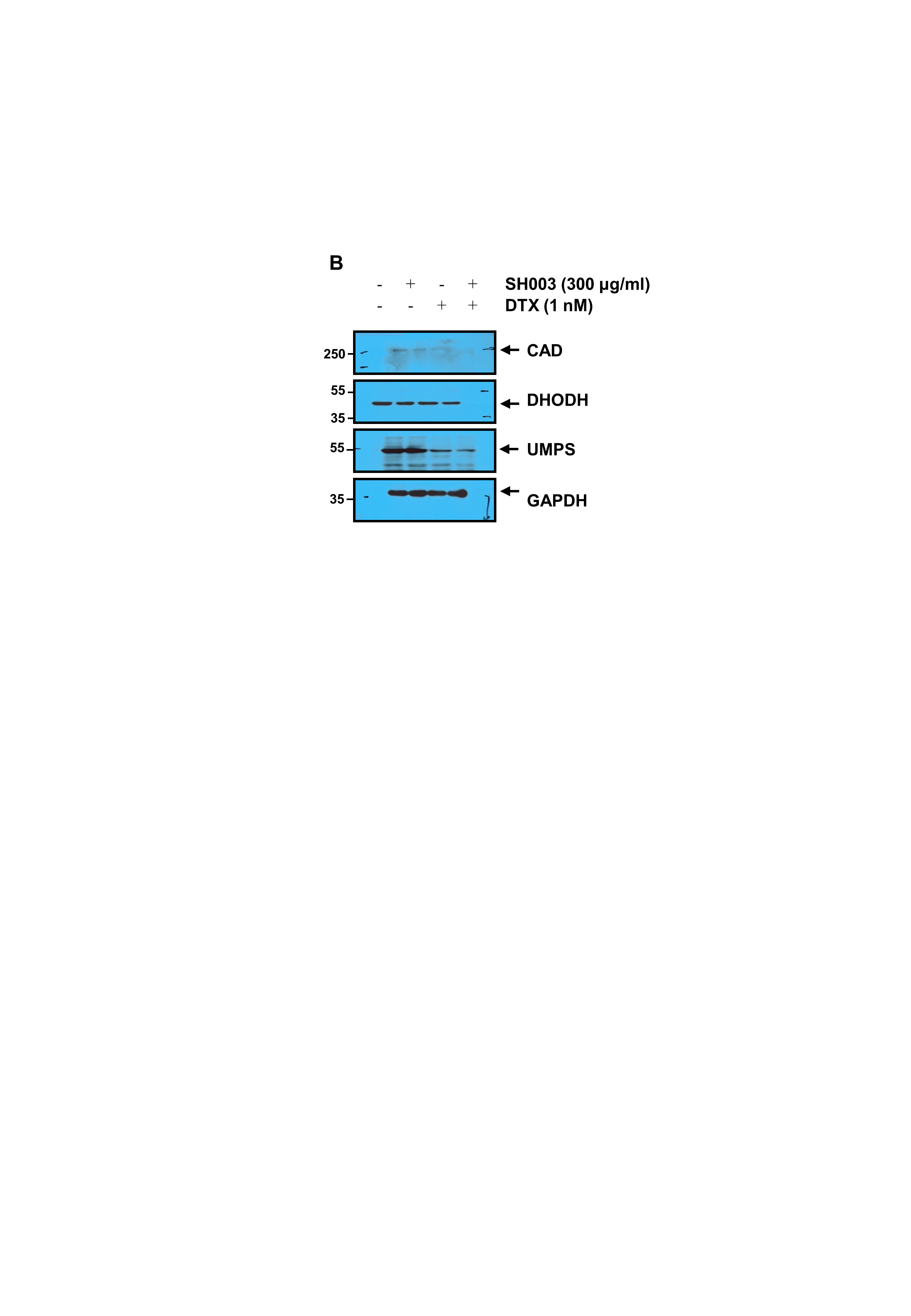

Supplement: Supplementary file 1 — Additional file 1. Uncropped western blot band in Figure 1 [file 12935_2024_3337_MOESM1_ESM.tif]

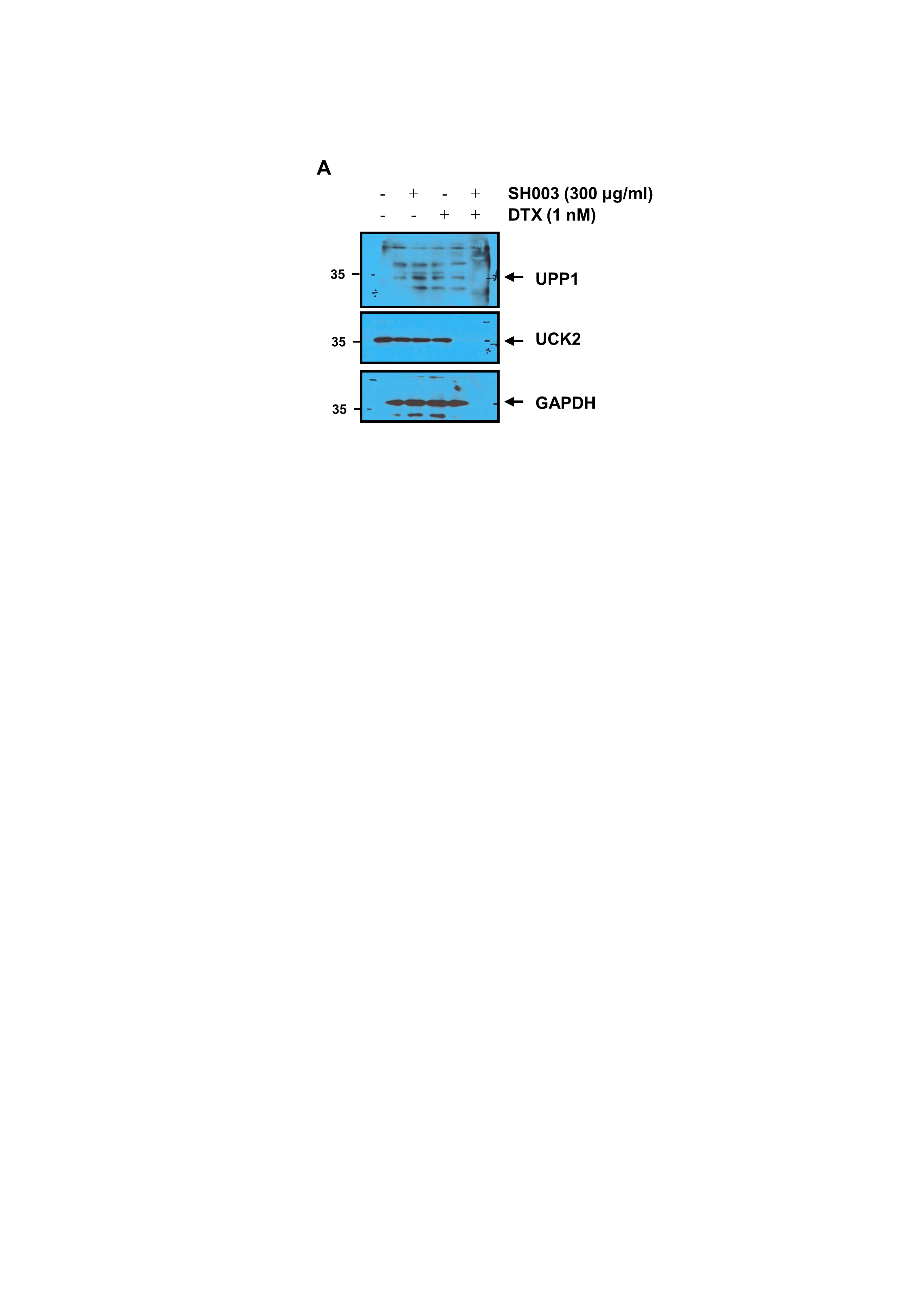

Supplement: Supplementary file 2 — Additional file 2. Uncropped western blot bands in Figure 2 [file 12935_2024_3337_MOESM2_ESM.tif]

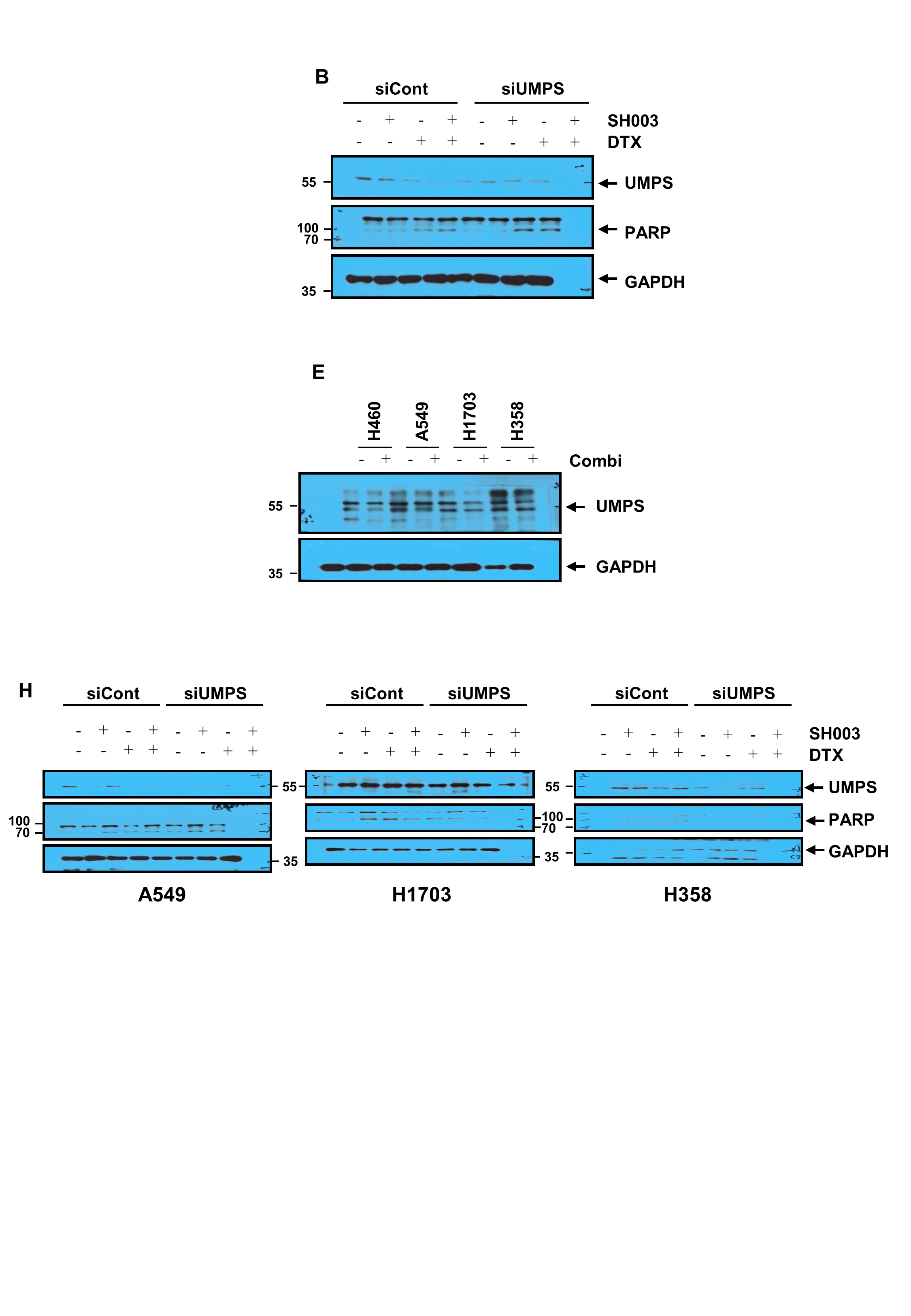

Supplement: Supplementary file 3 — Additional file 3. Uncropped western blot bands in Figure 3 [file 12935_2024_3337_MOESM3_ESM.tif]

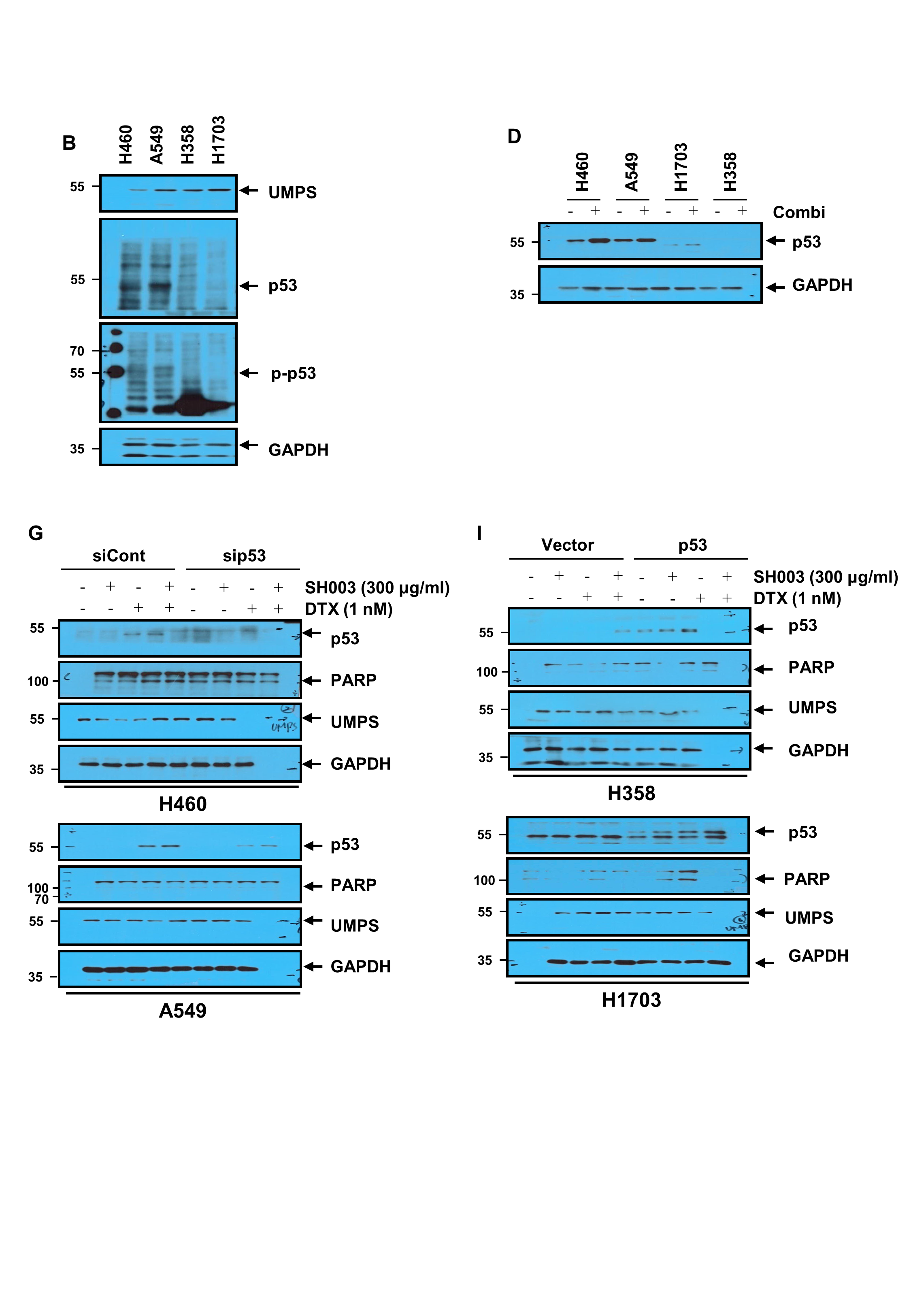

Supplement: Supplementary file 4 — Additional file 4. Uncropped western blot bands in Figure 4 [file 12935_2024_3337_MOESM4_ESM.tif]

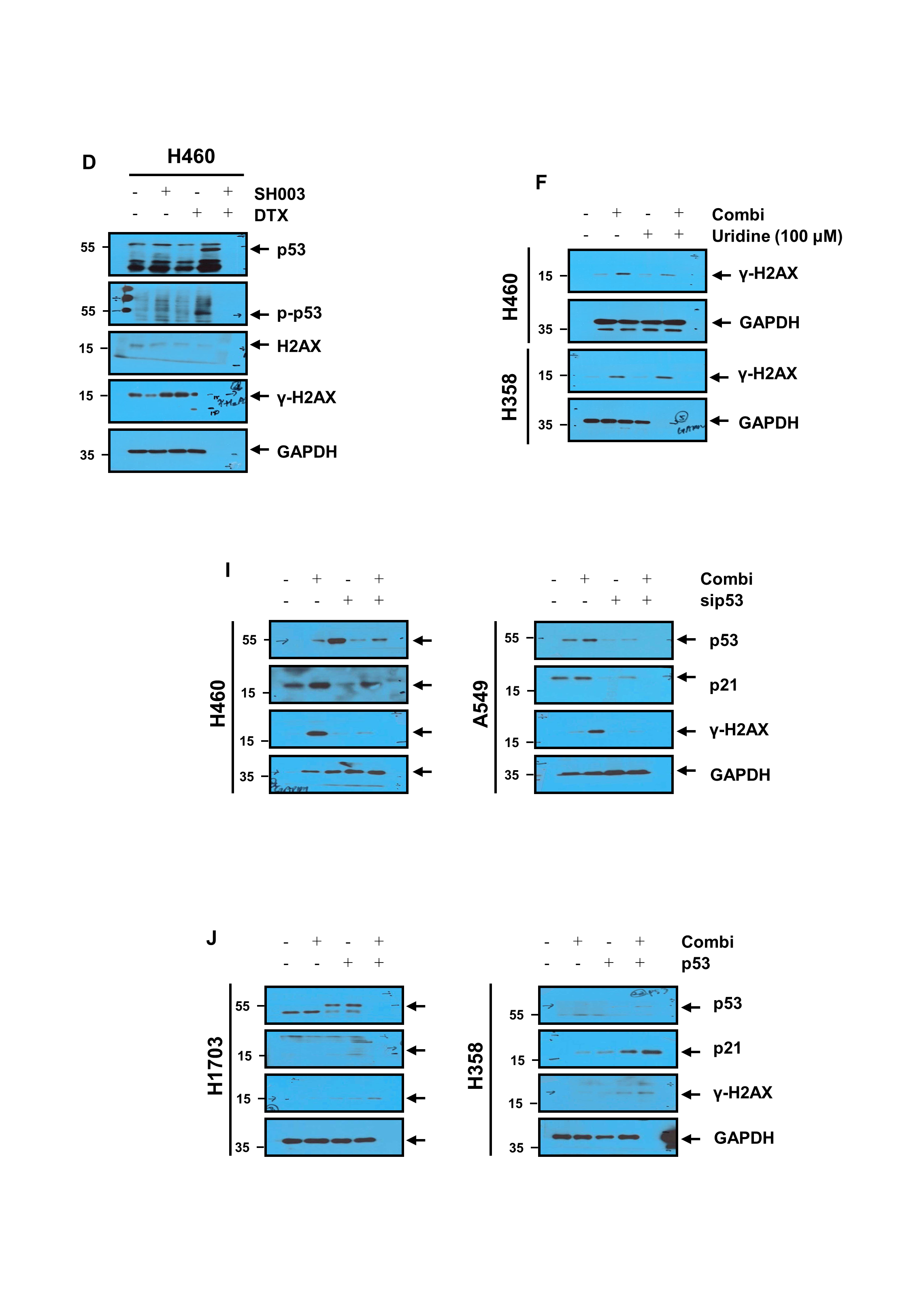

Supplement: Supplementary file 5 — Additional file 5. Uncropped western blot bands in Figure 5 [file 12935_2024_3337_MOESM5_ESM.tif]
